# Vesicular stomatitis forecasting based on Google Trends

**DOI:** 10.1371/journal.pone.0192141

**Published:** 2018-01-31

**Authors:** JianYing Wang, Tong Zhang, Yi Lu, GuangYa Zhou, Qin Chen, Bing Niu

**Affiliations:** Shanghai Key Laboratory of Bio-Energy Crops, School of Life Sciences, Shanghai University, Shanghai, P. R. China; Tampere University of Technology, FINLAND

## Abstract

**Background:**

Vesicular stomatitis (VS) is an important viral disease of livestock. The main feature of VS is irregular blisters that occur on the lips, tongue, oral mucosa, hoof crown and nipple. Humans can also be infected with vesicular stomatitis and develop meningitis. This study analyses 2014 American VS outbreaks in order to accurately predict vesicular stomatitis outbreak trends.

**Methods:**

American VS outbreaks data were collected from OIE. The data for VS keywords were obtained by inputting 24 disease-related keywords into Google Trends. After calculating the Pearson and Spearman correlation coefficients, it was found that there was a relationship between outbreaks and keywords derived from Google Trends. Finally, the predicted model was constructed based on qualitative classification and quantitative regression.

**Results:**

For the regression model, the Pearson correlation coefficients between the predicted outbreaks and actual outbreaks are 0.953 and 0.948, respectively. For the qualitative classification model, we constructed five classification predictive models and chose the best classification predictive model as the result. The results showed, SN (sensitivity), SP (specificity) and ACC (prediction accuracy) values of the best classification predictive model are 78.52%,72.5% and 77.14%, respectively.

**Conclusion:**

This study applied Google search data to construct a qualitative classification model and a quantitative regression model. The results show that the method is effective and that these two models obtain more accurate forecast.

## Introduction

Vesicular stomatitis is a highly contagious zoonotic infectious disease caused by the vesicular stomatitis virus (VSV). VSV is an RNA virus in the family Rhabdoviridae and genus Vesiculovirus, which includes the vesicular stomatitis virus New Jersey (VSV-NJ), Indiana (VSV-IN), and Alagoas serotypes (VSV-AV) and the Cocal virus [1]. New Jersey and Indiana are the two major serotypes of vesicular stomatitis. VSV-NJ belongs to the New Jersey serotype [2]. Vesicular stomatitis mainly affects cattle, sheep, camels and other ruminants. Approximately 10–15% of adult animals show clinical signs. Vesicular stomatitis is characterized by vesicles, papules, erosion and ulcer. These lesions mainly occur in the mouth or on the feet, teats and prepuce[3]. The disease varies by species. For example, blisters in horses usually appear on the upper surface of the tongue, lips and nostrils around the mouth and gums. Lesions in cattle occur mainly on the tongue, lips, gums, hard palate, and sometimes on the muzzle, and ulcers in pigs occur in the snout. Although these diseases do not cause death, they result in pain and anorexia in the animals. These diseases can give rise to secondary bacterial mastitis, which causes a loss of meat and milk production and seriously affects the development of animal husbandry [4]. Some vesicular stomatitis viruses also infect humans, although these infections are rare. Vesicular stomatitis has been classified as a grade A infectious disease by the World Organization for Animal Health (OIE) and as a class II infectious disease by the Animal Epidemic Prevention Law of the People's Republic of China [5].

Vesicular stomatitis mainly occurs in the Western hemisphere and is most common in the United States, Panama, Mexico, Peru and Venezuela. The United States has a higher prevalence rate of vesicular stomatitis than other countries. As early as 1926, the United States reported vesicular stomatitis in horses, followed by pigs, cattle and sheep [6]. Data from the United States Department of Agriculture (USDA) show that vesicular stomatitis outbreaks have continued in America since 2004, with vesicular stomatitis outbreaks in 2005 and 2014. In 2014, a vesicular stomatitis outbreak occurred in four American states (Arizona, Colorado, Nebraska, and Texas) [7].

Due to the danger of vesicular stomatitis, the need for an early warning system has received much attention. An early warning system has always been advocated as an important research field by some countries. Furthermore, an effective early warning system can significantly reduce the losses of the livestock industry [8]. However, traditional monitoring systems contain some defects, including inappropriate monitoring methods, data resource intensive, slow speed and difficulties with the collection of virus specimens [9]. Hence, the supplement and reform of traditional warning systems have become particularly significant. The appearance of new digital surveillance sources has brought new developments to animal epidemic monitoring, such as Google Trends.

Google Trends is a web-based tool for real-time surveillance of disease outbreaks that offers real-time information about the disease [10]. Currently, millions of people worldwide search online for health-related information every day, which makes web search queries a valuable source of information for the collection of health trends. In comparison with the network monitoring system, Google Trends shows great promise. The surveillance system offers timely, robust, and sensitive information and is widely used in disease surveillance research [11]. In recent years,“Google Trends” is realizing wider applications in epidemiological research. For example, Google Trends’ timely and accurate surveillance is commonly used in seasonal and pandemic influenza, which requires early detection of the outbreak [12–14]. The surveillance of other diseases, including Dengue, gastroenteritis, chickenpox and Tuberculosis, is also analysed by the near real-time search query data of Google Trends, which compensates for the deficiencies of traditional, healthcare-based, government-implemented surveillance [15–17].

The most common predictive early warning models include quantitative and qualitative models [18]. Multiple regressions are a quantitative model used to analyse the association between one independent variable and two or more dependent variables. They have been widely applied in epidemiology[19, 20]. For example, the seasonal autoregressive model was constructed using the SPSS software to analyse Hand-Foot-Mouth disease outbreaks [21]. Linear regressions and step regressions are quite commonly seen in the research of respiratory and cardiovascular diseases. They are also used to establish forecasting models for childhood colds and senile cerebrovascular diseases [22].

The classification forecasting model as a qualitative model has been extensively used in many fields, including medicine, epidemiology and molecular biology [23]. For example, Naïve Bayes with its unique strength was widely used in the forecasting system for heart disease. The detection system of cardiac arrhythmias within ECG signals was constructed using a Bayesian artificial neural network (ANN) classifier [24, 25].

In this study, the VS forecasting model was constructed using qualitative and quantitative approaches with data from OIE and Google Trends to predict the epidemic trend of vesicular stomatitis outbreaks. The keywords were selected from definitions and clinical symptoms of vesicular stomatitis. The trend data was available on Google Trends which include the numbers and geographical locations of searches [26].

We found that the keyword “vesicular stomatitis” in daily or weekly Google Trends had a higher relevance to outbreaks. After calculating the Pearson and Spearman correlation coefficients, 15 keywords that were positively correlated with outbreaks were chosen to construct a quantitative model. Thirteen keywords were used to build a classification forecasting model. Of those 13 keywords, 7 were negatively correlated with outbreaks and the remaining 6 keywords were randomly selected from 15 positively correlated keywords. The purpose of regression models is to use known Google Trends data to predict unknown outbreaks. The quantitative regression modelling method can illustrate the system’s development and direction with specific numerical values. Nine negative keywords cannot be used in the multiple linear regressions and multiple stepwise regressions since they are not compliant with the previous intentions of the regression models. However, all disease keywords represent real-time disease information and should not be ignored. When the relevance of the keywords is not good, the classification model can be used to analyse the disease outbreaks. Compared with regression models, the classification model is more tolerant to keywords. It accommodates almost all of the keyword-related information and is widely used in drug design and virtual screening [27]. Additionally, classification models have also achieved great success in disease monitoring [28]. Weka is a classification workbench for data mining that includes almost all mainstream classification algorithms [29]. AdaBoost is a supervised learning algorithm in Weka that has been applied as a very successful technique to solve the two-class classification problem [30]. Our approach uses AdaBoost to train a set of classifiers for outbreaks.

First, the vesicular stomatitis related keywords are individually filtered. After removing keywords that have a negative effect on the model, 13 keywords were selected to build a classification forecasting model. We constructed the classification model based on 5 classification thresholds and found that the classification model based on a threshold of 4 was optimal, with SN, SP and ACC values on the training set of 78.52%, 72.5% and 77.14%, respectively. The classification threshold was defined by vesicular stomatitis outbreaks. For example, if an outbreak data threshold of 4 is set as the boundary, then less than 4 cases are acceptable low frequency for outbreaks with 4 cases of tolerance. Outbreaks data for more than 4 cases are high frequency outbreaks, and urgent measures should be taken.

This study uses 2014 American vesicular stomatitis outbreaks as an example. First, the VS outbreak data were collected from the OIE. Then, Google search data were gathered by inputting disease-related keywords into Google Trends. Pearson and Spearman correlation analyses were performed between the disease outbreak and Google search data. Qualitative classification and quantitative regression models were constructed to predict vesicular stomatitis outbreaks and reduce the risk of disease.

## Data preparation

### Vesicular stomatitis outbreaks

The American vesicular stomatitis outbreaks from May 18th to November 25^th^, 2014 were coll-ected from the World Animal Health Information System (WAHIS) on OIE. The total number of VS outbreaks during that period was 433 [31]. (The data can be obtained from the link: http://www.oie.int/wahis_2/public/wahid.php/Reviewreport/Review/viewsummary?fupser=&dothis=&reportid=15320). The regression model was built with weekly data that was collected by setting s-unday as the first day of the week and daily data were collected to build a classification mod-el (see supplementary materials S1 and S2 Tables). All data collection is complied with the terms of service for OIE.

### Google Trends data

The keyword based Google Trends data were also divided into two types (weekly data and dai-ly data) and were collated from the Google Trends Platform. (The data can be obtained from the link: https://trends.google.com/trends/). The daily data were collated by keeping the dates in sync with the disease outbreaks date in OIE (May to December, 2014). The timeframe was li-mited to the whole of 2014 to obtain the weekly Google Trends data (see supplementary mat-erials S1 and S2 Tables). All data collection is complied with the terms of service for Google Trend-s.

### Keyword selection and correlation calculations

Twenty-four vesicular stomatitis related keywords were selected from the definitions and clinical symptoms of vesicular stomatitis [32]. From a comparison between keyword “vesicular stomatitis” Google Trends data and VS outbreaks, we deduced that there is a correlation between Google Trends data and outbreaks. Then, Pearson and Spearman correlation coefficients were calculated between each keyword's Google Trends data and the vesicular stomatitis outbreaks using IBM SPSS Statistics 20. The regression model was built with fifteen keywords (vesicular stomatitis, mouth ulcer, sore mouth, vesicular stomatitis virus, VSV, ulcer in mouth, inappetence, pyrexia, lameness, papules, ulcers, vesicular lesions, blister, blister lip, and lip blister) that were positively correlated with vesicular stomatitis outbreaks. The classification forecasting model was built using 13 keywords. Eight (mouth ulcer, sore mouth, ulcer in mouth, inappetence, pyrexia, lameness, blister lip, and lip blister) of the keywords were negatively correlated with outbreaks and the remaining 5 variables (vesicle, tongue blister, molar, excessive salivation, and lethargy) were randomly selected from the positively correlated keywords (see supplementary materials S1 Table).

## Methods

### Linear regression model

#### (1) Multiple linear regression and multiple stepwise regression methods

C was the number of outbreaks, and X1—X15 represented the 15 keywords that are positively correlated with outbreaks. A multiple linear regression model was constructed between the vesicular stomatitis outbreaks and the 15 disease-related keywords on the R 3.32 platform [33]. These 15 keywords are the independent variables used to predict the dependent variable (the number of vesicular stomatitis outbreaks). To optimize the multivariate the linear regression equation, a stepwise regression model was established to select the independent variables that had more influence on the dependent variables.

#### (2) Model prediction

The predicted outbreaks of the multiple linear regression and stepwise multiple regression methods were calculated after the model was assessed on R 3.32 [34]. Then, the correlation between the predicted value and VS outbreaks was calculated to compare the trend of the two sets of data and infer the accuracy of the model. The specific steps are as follows:

Input: S = {(C_i_, X_i_1_, X_i_2_, …, X_i_15_), _i = 1, 2, …, 28_}
Process:
Step1 // Multiple linear regression analysis on training set S
  Ms <- lm (C~X1+X2+X3+X4+X6+X7+X8+X9+X10+X11+X12+X13+X14+X15,S)
Step2 // The outbreaks prediction based on multiple linear regression
   ps <-predict (Ms, S)
Step3 // Stepwise regression
   Ss <- step (Ms)
Step4 // The outbreaks prediction based on stepwise regression
  Ps <-predict (Ss,S)
Output: Ps

### Classification forecasting model

#### (1) Data classification and variable screening

At first, a VS outbreak number of 1 was set as a threshold to classify the 13 keywords included in the 192 daily data sets from Google Trends in Excel (S3 Table). The Google search data corresponding to less than 1 outbreak were classified as A, and the data corresponding to greater than or equal to 1 outbreaks were classified as B. Second, the data set was randomly divided into a training set (175) and a test set (17). The training set was used to construct the training model, and the test set was used to evaluate the performance of the final model after each training run. Additionally, to avoid over-fitting, the test data set is used only once. The Google Trends data were classified for thresholds 2, 3, 4, and 5 using the same method used for threshold 1 (S4 and S5 Tables). The 13 keywords were filtered individually from the first keyword “vesicle”, and keywords that had a negative effect on the model were removed. The AdaBoost classifier in Weka 3.6.12 was chosen to construct the model [35, 36]. After deleting the variables that decreased the model’s sensitivity, specificity and accuracy, 13 keywords were significantly correlated with vesicular stomatitis outbreaks.

#### (2) Model construction and testing

The classification machine AdaBoost was combined with the trees, Bayes, functions, mi, misc, lazy, rules, meta and nested dichotomies functions to construct the threshold 1, 2, 3, 4, and 5 training set classification predicted models [37]. The modelling method with the best sensitivity (SN), specificity (SP) and accuracy (ACC) was selected [38]. Then, the test sets classified as thresholds 1, 2, 3, 4, and 5 were substituted into the constructed model, and the SN, SP and ACC values were calculated.

#### (3) AdaBoost

AdaBoost is the main classifier in our study and the classification model was constructed by combining AdaBoost with different weak classifiers. The processes of classic the AdaBoost algorithm (binary classification) are described below:

**Set sample set**:

$$D=\{\left(x_{1},y_{1}),(x_{2},y_{2}),\ldots ,(x_{m},y_{m})\right\}$$ (1)

$$x_{i}\in X,y_{i}\in Y=\{-1,+1\},i=1,\ldots ,m$$

#### Initialization

For each (*x*_*i*_, *y*_*i*_) ∈ *S D*_*t*_(*x*_*i*_, *y*_*i*_) = 1/*m*; AdaBoost repeatedly calls a given weak or basic learning classifier over a series of time intervals t = 1, 2,…, T, where *D*_*t*_(*x*_*i*_, *y*_*i*_) is the weight of sample (*x*_*i*_, *y*_*i*_) on the t_th_ cycle [39, 40].

1. We calling the WeakLearn program algorithm with parameter *D*_*t*_ and obtain the basic classification rule h_*t*_:X → Y. Namely, the classification rules of the t_th_ round are generated by the weak algorithm (Usually, a collection of rules).
2. We choose the correct α_*t*_ to describe the importance of *h*_*t*_ based on the measurement of the prediction error. α_*t*_ ∈ *R*, where α_*t*_ is the evaluation of classification rule h_*t*_ on round t. A larger α_*t*_, indicates a more important h_*t*_.
3. We generate the weight of each sample by running the algorithm to the (t+1)_th_ cycle. Incorrectly classified samples will have a greater weight on the t_th_ cycle. The specific weight update rule can be expressed as
  $$D_{t+1}(x_{i},y_{i})=\frac{D_{t}(x_{i},y_{i})∙e^{-\alpha_{i}y_{i}h_{i}(x_{i})}}{Z_{t}}$$ (2)
  where *Z*_*t*_ is the normalized constant. This results in
  $$\sum {}_{\left(x_{i},y_{i}\right)\epsilon S}\left(x_{i},y_{i}\right)=1$$ (3)

#### Output: The final classifier

Each round classification rule *h*_*t*_ acts on X such that

$$\text{H}\left(x\right)=sign\left(\sum_{t=1}^{T}\alpha_{i}h_{t}\left(x\right)\right)$$ (4)

## Results

### Correlation calculation and analysis

**Fig 1:**
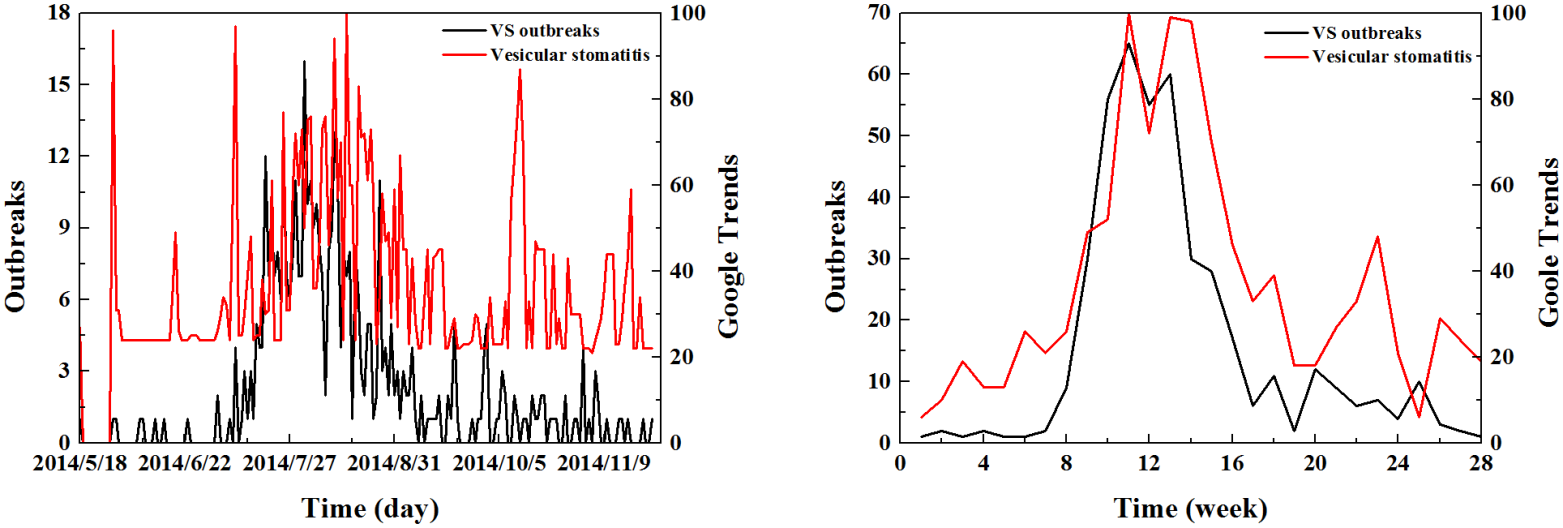
The trend for outbreaks and Google Trends data from the “vesicular stomatitis” keywords. (A) Daily Google Trends data. (B) Weekly Google Trends data.

After obtaining the 2014 American vesicular stomatitis outbreak data and the Google search data of the “vesicular stomatitis” keywords, the relationship of the two kinds of data was presented in the trend curve (Fig 1).

There is a correlation between VS outbreaks and Google Trends data from comparing these two sets of data. The outbreaks (black curve) and “vesicular stomatitis” keywords (red curve) are overlapped from June 22nd to August 31st, 2014 (using the daily Google Trends data). The trend of outbreaks (black curve) and “vesicular stomatitis” keywords (red curve) appear to be highly correlated especially from the fourth week to the twentieth week (using the weekly Google Trends data). Therefore, we can deduce that the Google Trends data for vesicular stomatitis keywords are correlated with vesicular stomatitis outbreaks. After collecting the 2014 American vesicular stomatitis outbreak data and the Google search data, Pearson and Spearman correlation analyses were performed between the 24 keywords, the weekly Google Trends data and the actual outbreak data. The results are shown in Table 1.

**Table 1:** The correlation coefficient value between the outbreaks and Google Trends data.

Parameter	Keywords	Correlation coefficient value	
		Pearson	Spearman
X1	vesicular stomatitis	0.853**	0.774**
X2	mouth ulcer	0.429*	0.215
X3	sore mouth	0.396*	0.208
X4	Vesicular Stomatitis Virus	0.387*	0.505**
X5	VSV	0.441*	0.566**
X6	ulcer in mouth	0.34	0.267
X7	inappetence	0.181	0.159
X8	pyrexia	0.151	0.013
X9	Lameness	0.145	0.11
X10	papules	0.349	0.113
X11	ulcers	0.187	0.264
X12	Vesicular lesions	0.228	0.149
X13	blister	0.337	0.075
X14	blister lip	0.244	0.045
X15	lip blister	0.255	0.077
X16	vesicle	-0.430*	-0.180
X17	gum blister	-0.061	-0.105
X18	tongue blister	-0.027	0.027
X19	molar	-0.489*	-0.298
X20	pruritus	-0.058	0.318
X21	anorexia	-0.442*	-0.306
X22	Sore nose	-0.365	-0.266
X23	excessive salivation	-0.133	-0.144
X24	lethargy	-0.234	-0.089

** Significant correlations at 0.01 level (bilateral)

* Significant correlation at 0.05 level (bilateral)

The correlation coefficient value from variables X1 –X15 are positive, indicating a positive interrelated relationship between vesicular stomatitis keywords and vesicular stomatitis outbreaks. The remaining vesicular stomatitis keywords (X16—X24) have a negative correlation with vesicular stomatitis outbreaks.

### Linear regression model

#### Regression parameter estimation

According to the R output, the multiple linear regression equation between the actual number of outbreaks Y and the vesicular stomatitis keywords X is:

Y=0.62930X1+0.08301X2−0.39479X3−0.22453X4+0.43043X5+0.10939X6+0.15337X7−0.02836X8−0.74344X9+0.70292X10+0.26618X11+0.55624X12+0.24883X13+0.13928X14−0.38455X15−113.49418.

The stepwise regression equation is:

Y=0.67343X1−0.35915X3+0.8829X10+0.26649X11+0.51229X12+0.20575X13+0.17802X14−0.52112X15−97.14635.

#### Significance test of the regression equation

To test the significance of individual and overall variables, the T test, F test, and R^2^ (coefficient of determination) were calculated in R. The multivariate linear regression T-test showed that only the independent vesicular stomatitis variable X1 was significant (***, Table 2). All of the variables were significant after the multiple stepwise regression analysis (Table 3).

**Table 2:** Significance test of multiple linear regression equations.

parameter	Std. Error	t value	value Pr(>|t|)
C	59.76067	-1.899	0.081845 .
X1	0.11353	5.543	0.000127 ***
X2	0.6045	0.137	0.89305
X3	0.39954	-0.988	0.342599
X4	0.37123	-0.605	0.55656
X5	0.40409	0.40409	0.307755
X6	0.32297	0.339	0.740694
X7	0.21058	0.728	0.480373
X8	0.10581	-0.268	0.793231
X9	1.10831	-0.671	0.515061
X10	0.53168	1.322	0.210789
X11	0.16166	1.647	0.125561
X12	0.35742	1.556	0.145608
X13	0.14746	1.688	0.117302
X14	0.15665	0.889	0.391436
X15	0.26838	-1.433	0.177433

Signif. codes: ‘***’ 0.001 ‘.’ 0.1

**Table 3:** Significance test of stepwise multiple regression equations.

parameter	Std. Error	t value	value Pr(>|t|)
C	33.84799	-2.87	0.0098 **
X1	0.08638	7.796	2.45e-07 ***
X3	0.20588	-1.744	0.0972 .
X10	0.43447	2.032	0.0564 .
X11	0.13101	2.034	0.0561 .
X12	0.23147	2.213	0.0393 *
X13	0.1026	2.005	0.0594 .
X14	0.11143	1.598	0.1266
X15	0.20456	-2.548	0.0197 *

Signif. codes: ‘***’ 0.001 ‘**’ 0.01 ‘*’ 0.05 ‘.’ 0.1 In the F and R^2^ tests, the P value of the stepwise multiple regression was 4.772e-07, which was less than the P value of 0.0005248 obtained in the multiple linear regression analysis. The stepwise multiple regression’s R^2^ was 0.8746 and the adjusted R^2^ was 0.8218, which were close to 1 (Table 4). An R^2^ is closer to 1 indicates that the majority of the dependent variable’s uncertainty can be explained by the regression equation, indicating a better goodness of fit. Based on the above results, the stepwise multiple regression results were superior.

**Table 4:** F test and R^2^ of regression model.

	P value	R^2^	Adjusted R^2^
Multiple linear regression	0.000525	0.8853	0.7617
Stepwise multiple regression	4.77E-07	0.8746	0.8218

### Model prediction

**Fig 2:**
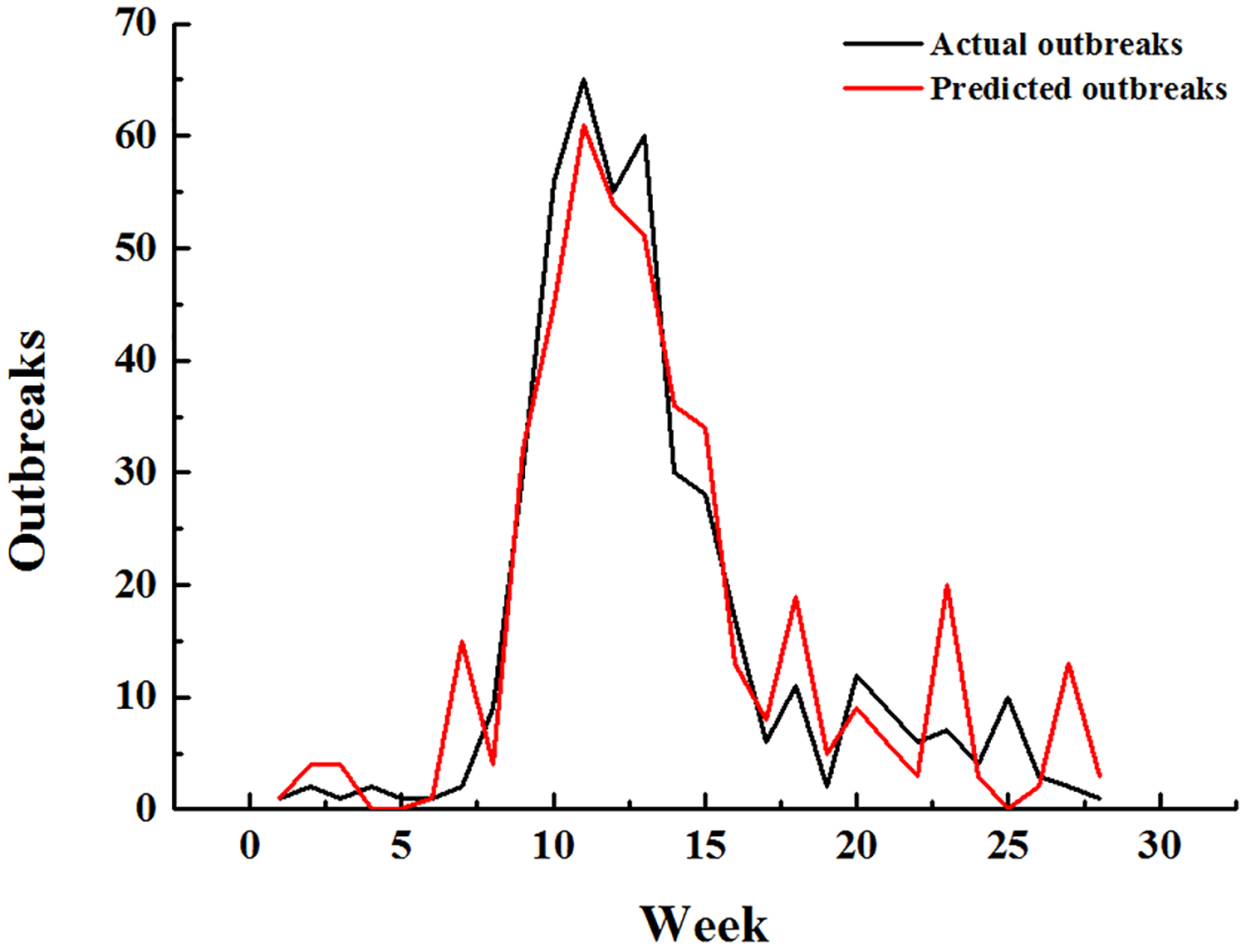
Comparison between the actual American vesicular stomatitis outbreaks and the predicted outbreaks using the multiple linear regression model.

**Fig 3:**
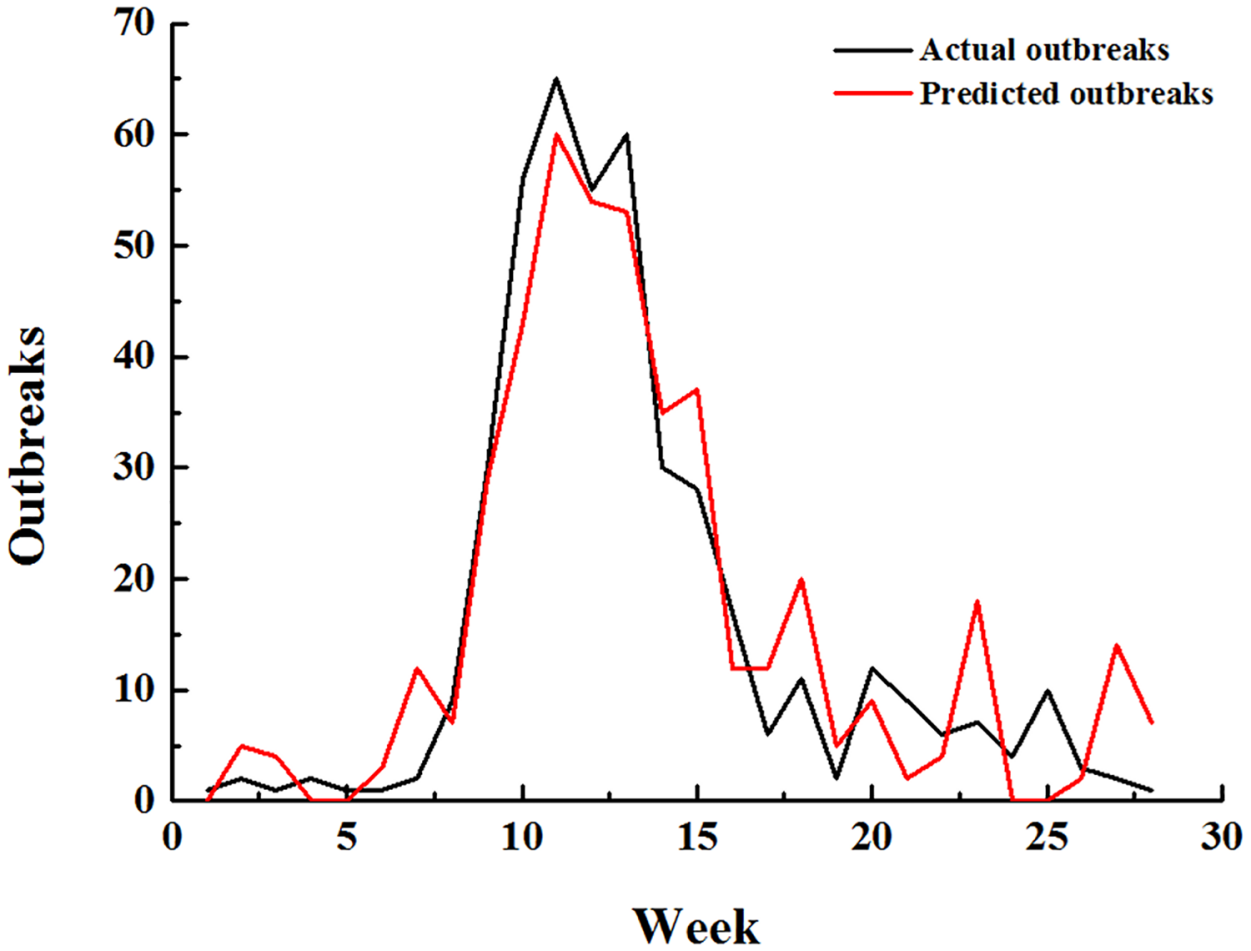
Comparison between the actual American vesicular stomatitis outbreaks and the predicted outbreaks using the stepwise multiple regression model.

We used the predict () function in R to predict the multiple linear regression and multiple stepwise regression data. The results are shown in Figs 2 and 3. The red curve (actual outbreaks) and the black curve (predicted outbreaks) have similar trends under the multiple linear regression and the multiple stepwise regression. The fit for the multiple linear regression is better than the multiple stepwise regression since the trend differences only appeared in the 23rd week in the multiple linear regression, while appeared in the 20th week in the multiple stepwise regression. The Pearson correlation coefficient calculations were conducted between the actual outbreaks and the predicted outbreaks. The results of the multiple linear regression and the multiple stepwise regression are 0.953 and 0.948, respectively.

### Classification model

The classifier AdaBoost was combined with different weak classifiers to construct the classification model. After inputting the training set of thresholds 1, 2, 3, 4, and 5, the model was constructed and tested with the independent test set. The classification model’s SN, SP and ACC from the training set exceeded 60% are shown in Table 5. In the 11 classification models, the prediction accuracy (ACC) of thresholds 3, 4 and 5 reached 70% and AdaBoost combined with DecisionStump of threshold 4 reached the highest value at 77.14%. The SP of the independent test set thresholds from thresholds 1 and 2 are under 50% and the model lacks stability. After the classification calculation, the model constructed by AdaBoost combined with DecisionStump from threshold 4 was found to be the best classification model, with SN, SP and ACC values of 78.52%, 72.5%, and 77.14%, respectively.

**Table 5:** AdaBoost combined with weak classifiers’ model.

Classification threshold	Classifier	Training set			Independent test set		
		SN(%)	SP(%)	ACC(%)	SN(%)	SP(%)	ACC(%)
1	NbTree	67.14	60	62.86	57.14	50	52.94
2	DecisionStump	71.17	60.94	67.43	81.82	33.33	64.71
	BayesNet	72.07	64.06	69.14	81.82	33.33	64.71
	MultiBoostAB	72.97	64.06	69.71	90.91	33.33	70.59
3	ComplementNaiveBayes	65.63	80.85	69.71	41.67	80.00	52.94
	NaiveBayesMultinomialUpdateable	76.56	61.7	72.57	66.67	80.00	70.59
4	DecisionStump	78.52	72.5	77.14	69.23	75.00	70.59
	ComplementNaiveBayes	61.48	95	69.14	46.15	75.00	52.94
5	NaiveBayesMultinomial	72.73	71.88	72.57	53.85	75.00	58.82
	NaiveBayesMultinomialUpdateable	71.33	68.75	70.86	53.85	75.00	58.82
	VIF	72.73	81.25	74.29	69.23	75.00	70.59

### Single variable predictions

The training and test sets were separately constructed using 4 as the classification threshold, and the integrated learning algorithm AdaBoost combined with DecisionStump was applied to examine the effect of a single variable on the model. The results are shown in Table 6. The ACC of the disease-related keywords exceeded 70%, indicating that the classifier AdaBoost combined with DecisionStump was a useful method for model classification. In addition, the ACC of each keyword is balanced, which means that there is no strong influencing factor. Therefore, the 13 keywords should be combined to construct a more accurate classification model.

**Table 6:** Single variable model constructed by AdaBoost+ DecisionStump.

Parameter	Training set		
	SN(%)	SP(%)	ACC(%)
X2	98.52%	7.50%	77.71%
X3	95.56%	0.00%	73.71%
X6	95.56%	0.00%	73.71%
X7	97.78%	2.50%	76.00%
X8	100.00%	0.00%	77.14%
X9	100.00%	0.00%	77.14%
X14	100.00%	0.00%	77.14%
X15	99.26%	0.00%	76.57%
X16	100.00%	0.00%	77.14%
X18	99.26%	0.00%	76.57%
X19	99.26%	0.00%	76.57%
X23	99.26%	0.00%	76.57%
X24	99.26%	0.00%	76.57%

## Discussion

A model is used to predict the future development of events of interest. The model is usually divided into two subsets, including (a qualitative prediction method and a quantitative prediction method) [41]. The qualitative analysis indicates a link between each disease keyword and VS outbreaks. In our study, the multiple stepwise regression results are almost the same as the disease outbreaks, with a correlation coefficient between the two variables of 0.948. Many keywords are associated with vesicular stomatitis, while not all of the Google search data are positively correlated with vesicular stomatitis outbreaks. Some disease keywords, such as “vesicles”, even presented a negative correlation. The inclusion of these keywords in the regression modelling process will affect the regression trend and be automatically deleted by the stepwise multiple regression process. However, all disease keywords represent real-time disease information and should not be ignored. When the relevance of the keywords is not ideal, the classification model with its tolerance can be used to analyse the disease outbreaks. At first, 21 vesicular stomatitis related keywords were used to construct the classification model by AdaBoost combined with combined with different weak classifiers in order to conceal the defects of other keywords. This was done after removing the keywords “vesicular stomatitis”, “vesicular stomatitis virus” and “VSV” that were strongly correlated with vesicular stomatitis outbreaks. However, the classification results using 21 keywords are not good. S8 Table (see supplementary materials S8 Table) shows that no accuracy (ACC) exceeds 60% for the classification models constructed by thresholds 1 and 3, and the ACC for thresholds 2, 4, and 5 are generally below 70%. We believe some keywords among the 21 vesicular stomatitis relative keywords have a negative impact on classification. Therefore, 21 vesicular stomatitis relative keywords were individually filtered. After removing the negative keywords, 13 keywords were selected to build a classification forecasting model. Seven of the keywords were negatively correlated with outbreaks and the remaining 6 keywords were randomly selected from the positively correlated keywords. The best classification model was AdaBoost combined with DecisionStump from classification threshold 4, with SN, SP and ACC values of 78.52%, 72.5%, and 77.14%, respectively. The classification threshold was defined by vesicular stomatitis outbreaks.

Classification models from thresholds 3, 4 and 5 are better than thresholds 1 and 2 since the uneven classification exists in the Google Trends keywords data. The unbalanced data set may affect the model’s accuracy. Due to the lack of an outbreak data set, the model is unable to provide any further classifications and thus warrants further exploration. The majority of American vesicular stomatitis outbreaks are 1 and 0. When we set a VS outbreak number as the threshold to classify the Google Trends data, the data of classes A and B obtained by the classification are not accurate. For example, the number in classes A from thresholds 1 is 78, while in threshold 5 it is 156 (S3 Table). The classified data is more balanced with thresholds 3 and 4.

In addition, the weak classifier combined with AdaBoost also played a very important role in the classification model’s construction. As a supervised learning algorithm, AdaBoost is used to correct the misclassified samples by increasing the weight so that the next iteration will focus on these samples and the classifier with higher accuracy, and the weight is also relatively higher after each iteration [42]. In this paper, the best classification model was obtained by using decision stumps as a weak classifier. Decision stumps are the simplest form of binary decision trees are often used as components ("weak learners" or "base learners") [43]. Decision stumps with one internal node make immediately connect the classification decision to the terminal nodes. The AdaBoost with the decision stump classifier acts as a feature selection mechanism to quickly and accurately make classification decisions. Moreover, AdaBoost with the weak classifiers NaiveBayesMultinomial, NaiveBayesMultinomialUpdateable and VIF also achieved good results. However, the classification model constructed by the common classifier BayesNet was not good and the ACC was 62.86%. Although BayesNet is widely used in various classification models due to its an ability to obtain the probability density functions (PDFs) of individual pattern classes from learning samples, it is not suitable for the samples in this study [44].

**Fig 4:**
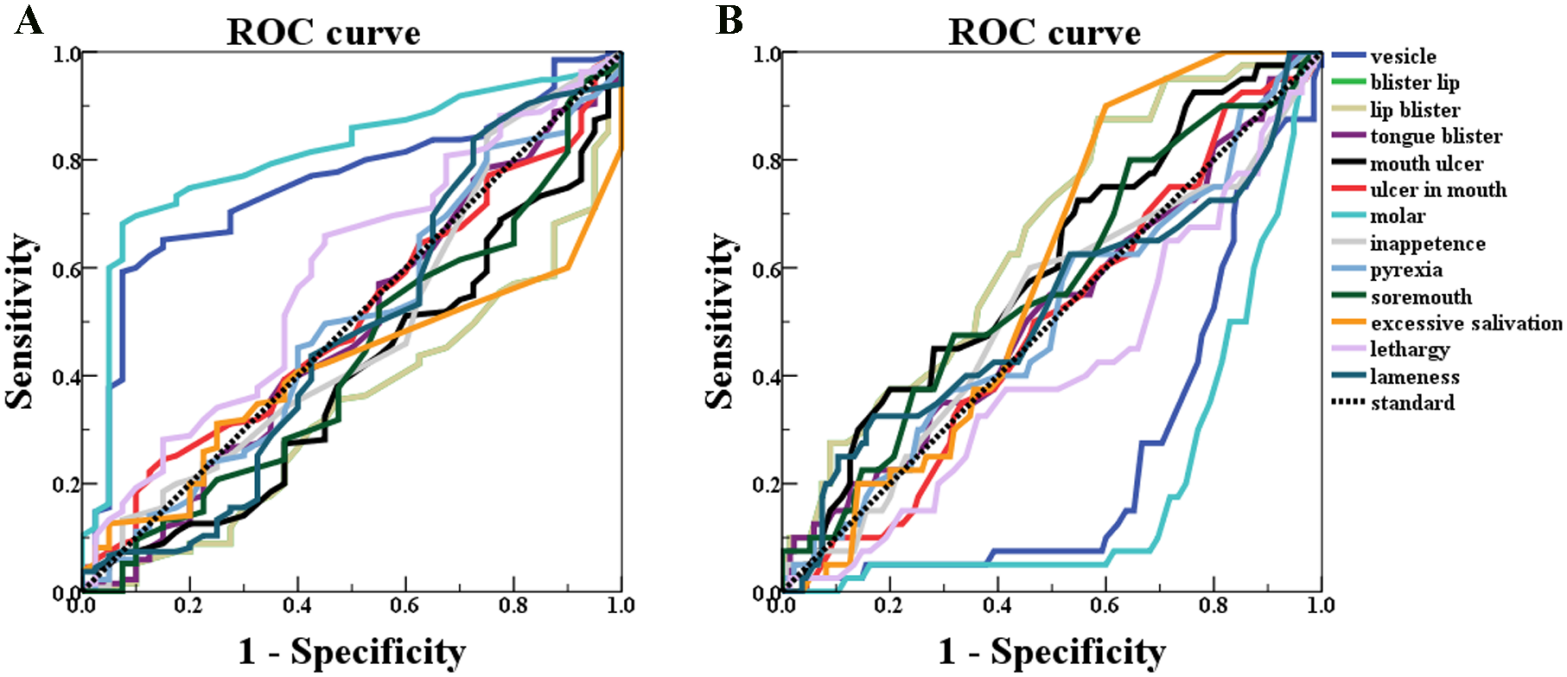
ROC curve of 13 keywords for vesicular stomatitis. (A) State variable is 0 (number of outbreaks<4) (B) State variable is 1 (number of outbreaks≥4).

The 13 keywords receiver operating characteristic (ROC) curve was created using SPSS to determine the most influential keywords in the model classification [45]. Fig 4(A) shows the ROC curve of the threshold 4 classification model. It can be easily concluded that keyword “Blister lip” (blue curve) is better than or at least as good as the others and “excessive salivation” (orange curve) (in Fig 4(B)) is the most influential keyword of Google Trends data classified by outbreaks greater than 4.

Traditional disease models are constructed using a single quantitative or qualitative method. For example, Chen Jinhong et al useda BP neural network to predict cardiovascular and cerebrovascular disease [46], Sang Youn Kim et al, used clinical decision support systems to predict advanced prostate cancer by comparing support vector machines and artificial neural networks [47]. In the United States, Berger used the Bayesian classification algorithm developed by the GIDEON system, which is a smart identification system for infectious diseases [48]. Feldman et al proposed using a decision tree technique on psychiatric diagnoses [49]. However, the two methods are rarely used to construct a disease prediction model. Therefore, the construction of one model is not complete due to the loss of accuracy and sensitivity.

In this study, the model was constructed using qualitative and quantitative approaches. Relevant keywords were used to construct a stepwise regression model and to explain the relationship between the disease keywords search data and the disease outbreaks. This model predicted the development and direction of vesicular stomatitis outbreaks. The classification model was constructed using negatively correlated keywords or keywords that were not significantly related to the disease to predict how many cases of VS were classed as high frequency outbreaks. This approach will allow the relevant departments to quickly take preventive measures. Although this article has made the most of the VS keywords, the search engine has some flaws. For example, the search engine cannot search for some keywords in Google Trends, resulting in the loss of some important disease information. In addition, 24 vesicular stomatitis related keywords were selected from the definition and clinical symptoms of vesicular stomatitis and should theoretically not be negatively correlated with VS outbreaks. The negative keywords are not compliant with previous regression models and cannot be used in multiple linear regressions or multiple stepwise regressions. There is still a need to combine more accurate information searches for disease information collection.

## Conclusion

In this work, we used two types of methods to construct the disease prediction model. The discovery of Google Trends is an important research area in mathematical modelling. As a timely, robust, and sensitive surveillance system, Google Trends is widely used in disease surveillance research. This study applied Google search data to construct a qualitative classification model and a quantitative regression model. The results show that the method is effective and that these two models obtain more accurate forecasting values.

## Supporting information

S1 Table Outbreaks and Google Trends data in week. (XLSX)

S2 Table Outbreaks and Google Trends data in day. (XLSX)

S3 Table The training and test set of thresholds 1. (XLSX)

S4 Table The training and test set of thresholds 2. (XLSX)

S5 Table The training and test set of thresholds 3. (XLSX)

S6 Table The training and test set of thresholds 4. (XLSX)

S7 Table The training and test set of thresholds 5. (XLSX)

S8 Table AdaBoost combined with weak classifiers’ model from 21 keywords. (XLSX)
